# Voluntary Contraceptive Uptake Among Postabortion Care Clients Treated With Misoprostol in Rwanda

**DOI:** 10.9745/GHSP-D-18-00399

**Published:** 2019-08-22

**Authors:** Catherine Packer, Allison P. Pack, Donna R. McCarraher

**Affiliations:** a FHI 360, Durham, NC, USA.; b Gillings School of Global Public Health, University of North Carolina at Chapel Hill, Chapel Hill, NC, USA.

## Abstract

**Introduction:**

Unsafe abortion remains a problem in Rwanda, where abortion is highly restricted by law. To reduce mortality and morbidity from unsafe abortion, Rwanda implemented a national postabortion care (PAC) program in 2012, which included using misoprostol to treat incomplete abortion. Key components of PAC are offering and providing voluntary contraceptive methods and counseling on their use, but little is known about contraceptive uptake among PAC clients treated with misoprostol. The objectives of the current study were (1) to assess the contraceptive uptake of PAC clients treated with misoprostol, including whether extended bleeding hinders uptake; and (2) to assess providers’ knowledge of contraception and their willingness to counsel PAC clients on contraception, provide methods, or refer for contraceptive services.

**Methods:**

We surveyed 68 PAC clients treated with misoprostol and 43 providers (84% nurses) in 17 health facilities across 3 districts in Rwanda where misoprostol for PAC had been introduced recently. PAC clients were recruited into the study prior to facility discharge and surveyed between 10 days and 1 month after discharge. We asked PAC clients and providers about demographic characteristics and attitudes toward contraception. We also asked PAC clients about contraceptive counseling received and postabortion contraceptive uptake or reasons for nonuse, and providers about their knowledge about return to fertility, pregnancy and contraceptive counseling, practices related to contraceptive method provision, and their knowledge and potential biases about PAC clients using contraception. We used descriptive statistics for analysis.

**Results:**

PAC clients were 19–46 years old, and most (69%) had at least 1 child. Almost all PAC clients (94%) reported being counseled on contraception, but only 47% reported choosing and receiving a method before being discharged from the facility. Nevertheless, by the time of the survey, 71% reported using a method. PAC clients’ main reason for not using contraception was wanting to become pregnant. Only 1 woman reported nonuse because of bleeding. Among providers, more than half (56%) reported there are contraceptive methods PAC clients should never use and about a quarter (26%) reported incorrect information on when PAC clients’ fertility could return.

**Conclusion:**

We found no evidence that bleeding associated with misoprostol for PAC influenced women's contraceptive uptake. However, as PAC programs expand to include misoprostol as a treatment option, accurate and high-quality postabortion contraception counseling and method provision at both treatment and follow-up visits must be strengthened.

## Abstract (precis)

Voluntary contraceptive uptake among postabortion care clients treated with misoprostol in Rwanda was high and unhindered by the extended bleeding that sometimes occurs with misoprostol use. However, provider knowledge regarding return to fertility and contraceptive methods appropriate for postabortion care clients should be strengthened.

## INTRODUCTION

Unsafe abortion remains a serious threat to women's health, especially in countries where abortion is illegal or highly restricted. In Rwanda, abortion is illegal except to save the life of a pregnant woman, or when the pregnancy is a result of rape, incest, or forced marriage or poses a risk to the health of the woman or the fetus.^1^^,^^2^ Although Rwanda's abortion law was amended in 2012 to reduce penalties and increase exceptions for permissible abortion, safe and legal abortion services remain extremely difficult to obtain due to burdensome processes, both women and health care professionals being unaware of the law, and/or stigma, leading many women to continue to resort to unsafe abortion.^1^^,^^2^ In 2009, 16,700 women aged 15–44 in Rwanda received care for complications resulting from unsafe induced abortion; however, about one-third of the women who experienced complications did not receive care.^1^ In response to this problem, Rwanda implemented a national comprehensive postabortion care (PAC) program beginning in 2012 to strengthen and expand services to reduce mortality and morbidity caused by unsafe abortion. Part of this comprehensive PAC program included introducing misoprostol as a treatment option that can be delivered in the communities where PAC clients live.

Incomplete abortion can be treated using surgical methods, such as vacuum aspiration or dilation and curettage (D&C), or nonsurgical medical methods, such as misoprostol. The World Health Organization (WHO) endorses the use of misoprostol as a first-line treatment for incomplete abortion, either induced or spontaneous (miscarriage), and several countries have introduced misoprostol for PAC.^3^^–^^6^ A single oral dose of 600 μg or a single sublingual dose of 400 μg effectively treats incomplete abortions and miscarriages.^7^ The advantages of using misoprostol over surgical methods to treat incomplete abortion include the following^7^:

- It can be administered by lower-level providers (e.g., nurses and midwives in primary health facilities), thereby increasing accessibility to those who need PAC.
- It is a “no touch” technique, which eliminates the need for surgical equipment, space, and highly trained personnel, and it takes providers less time than surgical techniques.
- It enables women to be more involved because the woman initiates the treatment.
- It is less invasive and feels more natural than a surgical procedure.
- It is inexpensive.
- It is easy to transport and store.
- It has few medical contraindications.

WHO endorses the use of misoprostol as a first-line treatment for incomplete abortion.

In addition, several clinical trials have reported misoprostol to be acceptable to women. Women who have used misoprostol report that they would use it again to treat an incomplete abortion or that they would recommend the use of misoprostol to other women in need of treatment for an incomplete abortion.^8^^,^^9^

Emergency treatment of incomplete abortion, miscarriage, and complications is only one component of PAC. It is equally important that PAC clients be counseled and offered contraceptive methods to prevent subsequent unintended pregnancies and repeat unsafe abortions. Several studies have illustrated significant increases in contraceptive uptake with modest intervention.^10^^–^^12^ However, these studies examined contraceptive uptake in settings where manual vacuum aspiration (MVA) and D&C were used for emergency treatment. We found only a single study that documented contraceptive uptake among PAC clients treated with misoprostol. In that 2010 study, operations research was conducted in Mozambique to document the feasibility and acceptability of misoprostol for treating incomplete abortion and miscarriage at all levels of the health system.^13^ Between July 2010 and January 2011, 300 PAC clients were treated with misoprostol. Service delivery data indicated that almost all PAC clients reported being counseled on contraception prior to facility discharge, and 81% chose and received a contraceptive method before leaving the facility. These methods included oral contraceptives (54.7%), injectable contraception (17.3%), and condoms (8.3%). The high rate of contraceptive uptake in Mozambique suggested that PAC clients treated with misoprostol were similar to those treated with MVA or D&C.

Emergency treatment of incomplete abortion, miscarriage, and complications is only one component of PAC. PAC clients should also be counseled and offered contraceptive methods.

However, contraceptive uptake among PAC clients treated with misoprostol might vary by setting and could be influenced by how well women tolerate the bleeding associated with misoprostol. One concern is that the extended bleeding associated with misoprostol might prevent women from initiating contraception. Other studies have documented that some PAC clients do not initiate contraceptive use after treatment because they believe they need to recover or heal.^14^^–^^16^ Moreover, bleeding irregularities are often cited as a reason why women discontinue method use in general.^17^^–^^19^

To advance our knowledge about voluntary contraceptive uptake among PAC clients treated with misoprostol in other contexts, we surveyed PAC clients and providers in 3 districts in Rwanda as a part of government-driven efforts to improve PAC that included the introduction of misoprostol as a treatment for postabortion complications.^20^ Our study objectives were to assess the contraceptive counseling and uptake among PAC clients treated with misoprostol, including whether extended bleeding associated with misoprostol affected contraceptive uptake, and to assess providers’ knowledge, attitudes, and practices of counseling PAC clients on contraception and return to fertility.

## METHODS

### Study Design and Setting

FHI 360 worked with the Rwanda Ministry of Health (MOH) and Venture Strategies Innovations (VSI) to conduct this cross-sectional study. In 2012, VSI was working with the Rwanda MOH to implement a comprehensive PAC pilot program that offered both surgical and medical methods (including the introduction of misoprostol) to treat postabortion complications in primary health care facilities and hospitals in 4 districts.^20^ In terms of misoprostol provision, providers (i.e., physicians, nurses, and midwives) were trained to administer misoprostol to eligible clients in stable condition with a gestational age of 12 or fewer weeks. Clients were to be given 3 tablets of 200 μg of misoprostol orally, stay at the facility for monitoring for 3 hours, and attend a follow-up visit 1–2 weeks after treatment. Providers were trained to provide counseling to PAC clients about all contraceptive methods, to talk to them about their reproductive goals, and to provide those who wanted to avoid pregnancy with condoms, pills, injectables, or implants or refer them for their method of choice prior to discharge at their treatment visit. Providers were trained that women could be offered an intrauterine device (IUD) at their follow-up visit. This study was built on VSI efforts to introduce misoprostol to treat incomplete abortion; at the time, MVA for PAC was only available at larger facilities. We implemented the study in 27 health facilities (district hospitals and health centers) that provide PAC in 3 of the 4 districts where the pilot program was being implemented: Gisagara, Bugesera, and Kicukiro districts.

Due to time and funding constraints, we had just over 1 month to recruit and survey study participants. We sought to survey as many PAC clients as possible during this time frame. PAC client eligibility criteria included being 15 years or older, receiving treatment for incomplete abortion at a study facility, and being willing to provide informed consent to participate in a survey. We trained study point persons at each facility to approach all PAC clients, during the recruitment time frame, after they received treatment (misoprostol, MVA, or D&C) for an incomplete abortion but prior to discharge, using a recruitment script to invite them to participate in the study. PAC clients who were willing to take part were asked to return to the facility to complete a survey between 10 days to 4 weeks after facility discharge. The 10-day minimum limit to the time frame allowed for some healing to take place, but also represents the minimal amount of time before a woman's fertility may return after experiencing a miscarriage and/or an incomplete abortion.

Eligibility criteria for providers included being involved in treating PAC clients at the study facility and being willing to give informed consent. We sought to survey at least 1 PAC provider at each facility where PAC clients were enrolled. In facilities with multiple PAC providers, more than 1 provider was invited to take part in a survey if they were available. We administered surveys to PAC providers (defined as a nurse, doctor, or midwife who provides medical treatment, counseling, or both to a PAC client) on the same days PAC clients were surveyed.

### Data Collection

We developed surveys to obtain information to answer our study objectives and pretested them prior to data collection. From March to April 2012, trained female Rwandan interviewers administered surveys in Kinyarwanda to PAC clients and providers using personal digital assistants (PDAs) to electronically record participants’ responses. We asked clients about demographic and reproductive health characteristics, attitudes toward contraception and previous contraceptive use, contraceptive counseling received during their initial treatment and follow-up visits, and postabortion contraceptive uptake or reasons for nonuse. We asked providers about their demographic characteristics, attitudes toward contraception, knowledge about return to fertility, pregnancy and contraceptive counseling, practices related to contraceptive method provision, and their knowledge and potential biases about PAC clients, including young women, using contraception.

### Data Analysis

The study coordinator from FHI 360 Rwanda transmitted the survey data to FHI 360 North Carolina using a secure server. We imported the data from PDAs into SAS software version 9.3^21^ and used descriptive statistics for analysis. We restricted the client analysis to PAC clients treated with misoprostol since only 2 were treated with D&C and none were treated with MVA. We restricted the provider analysis to providers who had provided any PAC in the prior 3 months. We translated open-ended responses that had been typed into the PDAs from Kinyarwanda to English and categorized and summarized the responses. Two analysts from FHI 360 North Carolina independently produced all tables and resolved discrepancies.

### Ethical Considerations

This study was reviewed and approved by FHI 360's Protection of Human Subjects Committee and the Republic of Rwanda National Ethics Committee. All PAC clients and providers gave written informed assent (ages 15–20 years) or informed consent (age 21 years and above) prior to completing a survey. After the survey, we reimbursed PAC clients about US$3.40 for their travel expenses per local norms. We did not compensate providers for participating because we conducted the survey at their workplace; therefore, no travel was involved.

## RESULTS

### PAC Client Characteristics

In total, 70 of 77 PAC clients who agreed to participate returned to the facility and completed the survey. We excluded from this analysis 2 PAC clients treated with D&C, resulting in a total analysis sample of 68 PAC clients who were treated with misoprostol. We surveyed the PAC clients at 17 facilities, including 4 district hospitals (n=33) and 13 primary care health centers (n=35). During the recruitment period, 10 of the study facilities had no PAC clients. The mean age of the PAC clients was 29.5 years (range 19–46), the majority had attended at least some primary school, and most were married (Table 1). About 70% reported having at least 1 living child and having used contraception previously. Study participants reported that they had primarily used short-acting contraceptive methods in the past, including injectables (53%), oral contraceptives (28%), and male condoms (7%). Few women had experience with long-acting methods; only 2 women reported that they had used implants previously. Eleven women (16%) reported that they were using a method at the time they became pregnant, including oral contraceptives, injectables, male condoms, CycleBeads, and withdrawal (data not shown). The need for postabortion contraception was great, with 60% of the women reporting that they wanted to delay childbearing and 16% reporting that they wanted to forgo all future childbearing. Finally, 13% of the women reported that they did not think they could get pregnant in the future.

Study participants reported that they had primarily used short-acting contraceptive methods in the past.

**TABLE 1.** PAC Client Sociodemographic and Reproductive Health Characteristics (N=68)

	Value
**District, No. (%)**	
Gisagara	29 (43)
Bugesera	28 (41)
Kicukiro	11 (16)
**Number of PAC clients by facility type, No. (%)**	
District hospital (4)	33 (49)
Health center (13)	35 (51)
Age, years, mean (range)	29.5 (19, 46)
**Highest educational level attended, No. (%)**	
Did not attend school	7 (10)
Primary	53 (78)
Secondary	8 (12)
**Has a paid job, No. (%)**	4 (6)
**Marital status, No. (%)**	
Currently married	50 (74)
Not married but living with partner	14 (21)
Not married but has boyfriend	2 (3)
Single, not in union	2 (3)
**Has living child(ren), No. (%)**	47 (69)
**Has ever used a contraceptive method, No. (%)**	48 (71)
**Prior contraceptive method used, No. (%)**	
Oral contraceptives	19 (28)
Injectables	36 (53)
Male condoms	5 (7)
Implants	2 (3)
CycleBeads	1 (1)
**Was using method when became pregnant, No. (%)**	11 (16)
**Future pregnancy plans, No. (%)**	
Immediately	6 (9)
Wait to become pregnant	41 (60)
Never want to become pregnant again	11 (16)
Do not think can get pregnant	9 (13)
Do not know	1 (1)

Abbreviation: PAC, postabortion care.

### PAC Clients’ Reports of Contraceptive Counseling and Uptake

Initiating contraceptive use is predicated on women understanding their risk of pregnancy postabortion. Among the PAC clients surveyed, 40% reported that they were not told when they could become pregnant again while 49% reported being told that they should delay pregnancy for at least 6 months (Table 2). However, 94% of the PAC clients reported that they were counseled on contraceptive methods.

**TABLE 2.** PAC Client Contraceptive Counseling and Uptake (N=68)

	No. (%)
**Client counseling**	
Not told when they could become pregnant again	27 (40)
Counseled to wait at least 6 months before becoming pregnant	33 (49)
Counseled on voluntary family planning	64 (94)
**Contraceptive uptake**	
Chose and received method before facility discharge	32 (47)
Using method at time of survey	48 (71)
**Method using at the time of the survey**^a^	
Injectable	22 (46)
Oral contraceptives	10 (21)
Male condom	9 (19)
Implant	3 (6)
CycleBeads	2 (4)
Missing	2 (4)
**Reasons for nonuse**^b^	
Wanted to get pregnant	6 (30)
Thinks unable to get pregnant	3 (15)
Disapproves of family planning	2 (10)
Believes body is weak/needs to recover	3 (15)
Reports family planning not available	2 (10)
Has infrequent sex or is no longer with partner	1 (5)
Worries that bleeding is too much	1 (5)

Abbreviation: PAC, postabortion care.

a N=48.

b N=20.

Almost half (47%) of the women said they chose and received a contraceptive method after being treated at their initial visit, prior to facility discharge. At the time of the survey, 71% of the women reported that they were using contraception. Those who were using a method at the time of the survey most likely obtained contraception from another source, such as a secondary health post or pharmacy; however, the survey did not capture this information. Most PAC clients reported using short-acting methods, including injectables, oral contraceptives, and condoms, at the time of the survey. Only 3 were using the implant, and none reported using an IUD. The PAC clients gave a variety of reasons for not using a contraceptive method, including the desire to become pregnant again, the belief that they were unable to get pregnant, disapproval of contraception, and concern that their bodies needed time to recover. Only 1 woman reported that she was not using a method because she was concerned about the bleeding she was experiencing.

Almost half of the women said they left the facility with a contraceptive method, and at the time of the survey, 71% reported they were using contraception.

### PAC Provider Characteristics

We surveyed a total of 47 PAC providers; however, 4 providers had not provided any PAC in the past 3 months. Those providers were excluded from this analysis, resulting in a total analysis sample of 43 providers. PAC for treatment of incomplete abortion could include misoprostol, MVA, or D&C. We surveyed providers from 16 different facilities, including 4 district hospitals and 12 health centers. We surveyed at least 1 provider from all but 1 health facility where we surveyed PAC clients. Most providers were nurses (84%) while a few were midwives (9%) and physicians (7%) with varying levels of experience treating PAC clients (Table 3). In total, 72% of providers reported that they attended the Rwanda MOH/VSI training on using misoprostol to treat PAC clients.

**TABLE 3.** PAC Provider Sociodemographic Characteristics and PAC Experience (N=43)

	No. (%)
**District**	
Gisagara	19 (44)
Bugesera	19 (44)
Kicukiro	5 (12)
**Number of providers by facility type**	
District hospital (4)	17 (40)
Health center (12)	26 (60)
**Sex**	
Male	15 (35)
Female	28 (65)
**Age, years, mean (range)**	32 (25–52)
**Job title**	
Nurse	36 (84)
Physician	3 (7)
Midwife	4 (9)
**Length of time providing PAC**	
<6 months	12 (28)
6 months to 1 year	3 (7)
1–5 years	15 (35)
>5 years	13 (30)
**Participated in VSI/RMOH training on misoprostol for PAC**	31 (72)

Abbreviations: PAC, postabortion care; RMOH, Rwanda Ministry of Health; VSI, Venture Strategies Innovations.

### PAC Providers’ Reports of Contraceptive Counseling and Method Provision

All providers surveyed reported that in the past 3 months they had talked with PAC clients about the clients’ desire to have children in the future (Table 4). However, some providers were misinformed about when PAC clients could become pregnant again, with 26% indicating that they could not get pregnant for a month or more after treatment. Most providers (93%) reported that they advised PAC clients to wait 6 months before becoming pregnant again. All providers reported that in the past 3 months they had counseled PAC clients on contraception or referred them to a secondary health post (a lower-level facility that provides contraceptive methods). Providers reported that they typically discussed a variety of methods with PAC clients; however, more providers reported discussing short-acting methods, such as oral contraceptives and injectables (84% and 91%, respectively), than long-acting methods, such as IUDs and implants (65% and 77%, respectively). Only 56% of providers reported that they discussed male condoms with PAC clients.

**TABLE 4.** PAC Providers’ Experience Providing Contraceptive and Pregnancy Counseling (N=43)

	No. (%)
**Talked to PAC clients about future plans to have children**^a^	43 (100)
**Thinks PAC clients can become pregnant again:**	
Within 10 days of treatment	28 (65)
After 1 month or more post treatment	11 (26)
**Tells PAC clients to wait 6 months before becoming pregnant again**	40 (93)
**Feels has enough time to counsel/refer PAC clients to voluntary contraception**	29 (67)
**Counseled PAC clients on voluntary contraception/referred them to secondary health post for method**^a^	43 (100)
**Discusses long-acting and permanent methods**	
IUD	28 (65)
Implants	33 (77)
Female sterilization^b^	8 (19)
Vasectomy	7 (16)
**Discusses short-acting methods**	
Oral contraceptives	36 (84)
Injectables	39 (91)
Male condoms	24 (56)
Female condoms	13 (30)
Emergency contraception pills	4 (9)
**Discusses fertility awareness methods**	
CycleBeads^c^	9 (21)
**Provider personally gives contraceptive methods to PAC clients**	32 (74)

Abbreviation: PAC, postabortion care.

a In the past 3 months.

b PAC clients are generally asked to return to the facility to seek this procedure.

c This method cannot be initiated by a PAC client until return to normal menses; this is important to avoid a future unintended pregnancy.

About one-third of providers reported feeling that they generally did not have enough time to provide contraceptive counseling or referrals to PAC clients. Approximately three-quarters of providers said that they personally give PAC clients contraceptive methods. Providers’ main reason for not personally giving clients contraceptive methods was that methods were not available where PAC was delivered, or that they were not dispensed in the facility at all (data not shown). Providers thought that the main reason PAC clients do not use contraception was that they want to become pregnant again (data not shown).

### PAC Provider Knowledge and Biases

Providers’ willingness to counsel PAC clients on contraception could be influenced by their knowledge of which methods PAC clients can use and their personal opinions on providing women and young women information on contraception.^14^^,^^15^^,^^22^ More than half (56%) of providers reported that there were some contraceptive methods that PAC clients should not use, with 37% reporting that PAC clients should not use IUDs (Table 5). When we investigated biases PAC providers might have toward unmarried and young women, we found that almost all PAC providers believed that unmarried women should not have sex and 42% believed that giving contraception to young women would motivate them to have sex.

More than half of providers reported that there were some contraceptive methods that PAC clients should not use.

**TABLE 5.** PAC Providers’ Postabortion Contraceptive Knowledge and Opinions (N=43)

	**No. (%)**
**Reported there are methods that should never be used by PAC clients**	24 (56)
**Methods believed should never be used by PAC clients**	
IUD	16 (37)
Implants	1 (2)
Injectables	1 (2)
CycleBeads	4 (9)
Female sterilization	2 (5)
Any traditional method	1 (2)
No response	1 (2)
**Agreed with statement: “Unmarried women should not have sex until marriage.”**	41 (95)
**Agreed with statement: “Giving family planning to PAC patients under 20 years old will motivate them to have sex.”**	18 (42)

Abbreviation: PAC, postabortion care.

## DISCUSSION

This study is one of the first to explore PAC client and provider perspectives on contraceptive uptake among clients treated with misoprostol for PAC. In this study, almost all surveyed PAC clients in 3 selected districts of Rwanda reported being counseled on contraception, almost half reported that they chose and received a contraceptive method prior to facility discharge, and nearly three-quarters reported that they were using a method at the time of the survey. We found no evidence to suggest that the bleeding associated with misoprostol use inhibited PAC clients’ contraceptive uptake. All PAC providers reported counseling women on contraception; however, they were more likely to counsel and provide PAC clients with short-acting rather than long-acting methods. Providers did not consistently have or deliver accurate information on return to fertility, some had misconceptions about what contraceptive methods PAC clients could use, and several held negative opinions toward unmarried women having sex and young women being given contraception.

We found no evidence to suggest that the bleeding associated with misoprostol use inhibited PAC clients’ contraceptive uptake.

The levels of contraceptive uptake we found for PAC clients treated with misoprostol in 3 districts in Rwanda were similar to the results of studies that examined contraceptive uptake after strengthening postabortion contraception services (e.g., providing contraception at the same time and location as PAC) for women treated with surgical methods in other African countries, such as Burkina Faso (83%), Ethiopia (78%), and Tanzania (70%).^23^ Our results are also similar to an evaluation of the pilot program implemented by the Rwanda MOH with support from VSI. That evaluation, done in 2013, reported that more than 80% of PAC clients were treated with misoprostol and 59% chose and were discharged with a contraceptive method.^20^ Our results indicate that postabortion contraceptive uptake of women treated with misoprostol for incomplete abortion is comparable with the contraceptive uptake of women treated with other methods for incomplete abortion and is not hindered by bleeding associated with misoprostol.

The main strength of our study is that we surveyed women after they were discharged from the health care facility. This gave PAC clients time to start using the method they were discharged with or to seek a method from another location and start using it. This is especially important in Rwanda because facilities with a religious affiliation, predominantly Catholicism, do not provide methods on site but are trained to refer PAC clients to secondary health posts located nearby for contraception.^20^ Several of our study facilities were Catholic and did not offer modern contraceptive methods. Therefore, between facility discharge and the survey, PAC clients likely went to a secondary health post or another location (e.g., pharmacy) to obtain a contraceptive method, which may explain the gap between facility discharge with a method and reported contraceptive use at the time of the survey. It should be noted that the amount of time to adopt contraception varied since clients were interviewed between 10 days to 4 weeks after receiving PAC. It is also important to note that some clients may have sought PAC due to miscarriage, which may explain why the main reason for contraceptive nonuse was the desire to become pregnant again.

Like previous studies,^24^ our study revealed that providers were more likely to counsel and provide PAC clients with short-acting rather than long-acting or permanent methods. Most PAC clients in this study who were using contraception at the time of the survey reported using injectables, oral contraceptives, and male condoms. Very few reported using implants, and none were using IUDs. These findings are similar to the contraceptive use reported among all women in the 2010 Rwanda Demographic and Health Survey (the time period closest to our survey) in which the most commonly used modern contraceptive methods were injectables (14.6%), oral contraceptives (3.9%), implants (3.6%), and male condoms (2.9%).^25^ Moreover, only 0.2% were using IUDs. Long-acting methods should be offered to PAC clients because they are highly effective in reducing unintended pregnancies and are appropriate for most PAC clients. According to WHO guidelines, IUDs can be safely used by PAC clients, but those treated with misoprostol should not get an IUD inserted until a follow-up appointment to ensure complete abortion.^3^ Although PAC providers in Rwanda were trained to offer IUDs to women treated with misoprostol for PAC, more than one-third of providers in our study felt that IUDs should never be used by PAC clients. In practice, few providers actually provide postabortion IUDs. In addition, some women, especially young women, do not attend follow-up appointments due to inconvenience or fear of stigma related to abortion.^5^^,^^26^ Therefore, more work should be done to explore strategies to overcome challenges in promoting postabortion IUD uptake. Same-day implant insertion for PAC clients who want to prevent pregnancy should also be encouraged since this is an increasingly popular method in Rwanda.^27^

Long-acting methods should be offered to PAC clients because they are highly effective in reducing unintended pregnancies and are appropriate for most PAC clients.

Our study found that PAC providers did not deliver information on return to fertility to all PAC clients. Further, not all providers had accurate information on it. Women receiving PAC need accurate information on when they can become pregnant again in order to make informed contraceptive choices. In addition, although almost all providers said they counseled women to wait 6 months to become pregnant again, just under half of PAC clients reported receiving this advice. Providers may have overreported this, or perhaps they counseled some but not all PAC clients. Furthermore, almost all PAC providers in this study felt that unmarried women should not have sex and almost half felt that giving young women contraception would motivate them to have sex, which likely influenced the way they counseled PAC clients. Enhanced training for providers on accurate information about return to fertility and which methods can safely be used by PAC clients, coupled with stronger referrals to contraceptive services from facilities where contraception is not available, will likely further increase contraceptive uptake. A key consensus statement by major international donors and health provider associations on postabortion voluntary family planning recommends providing PAC clients with skilled counseling to create a plan for obtaining ongoing contraceptive supplies as well as simple written instructions and information about their method of choice.^28^

Nearly half of pregnancies in Rwanda are unintended,^1^ and nearly one-fifth of women are not using modern contraception but wish to delay, space, or limit childbearing.^27^ Rwanda has made significant progress in improving reproductive health in recent years. Since this study was conducted, integration of comprehensive PAC into other services has continued; however, work remains to be done, specifically adding misoprostol for PAC to the list of essential medicines at the health center level, updating the comprehensive PAC guidelines, and continuing to have more practical trainings for providers to strengthen their PAC skills. Therefore, contraceptive services—and postabortion contraception services in particular—must continue to be strengthened to help women avoid future unintended pregnancies.

Voluntary contraceptive services—and postabortion contraception services in particular—must continue to be strengthened to help women avoid future unintended pregnancies.

### Limitations

This study had some limitations. The main limitation is that our small sample size only allowed presenting our findings descriptively and in aggregate. Consequently, we were unable to make meaningful comparisons between districts, types of health facilities, and younger versus older women. In addition, given that we relied on self-reported data, our results are subject to recall and social desirability biases. It is unknown whether these biases would inflate or underestimate the percentage of women who said they chose and were discharged with contraception and were using it at the time of the survey. These biases may have inflated providers’ responses about counseling women on contraception and return to fertility. Finally, this study included only women who obtained PAC and agreed to be interviewed; we do not know if or how the perspectives and contraceptive uptake might differ for women who did not seek PAC or refused to be interviewed.

In general, women aged 24 years or younger are more likely to have unsafe abortions than older women, especially in Africa^29^; however, few PAC clients in our study reported being 24 years or younger. It is possible that PAC clients in this study may have reported being older and/or married due to the strong stigma against young people using contraception and abortion in general in this context. This may have led to an undercount of younger women and adolescents. Moreover, adolescents are less likely to seek PAC,^26^ which may have affected the number of young women available to participate in this study. Future research should focus on documenting the experiences and perspectives of adolescent and young PAC clients since they may differ from experiences of older women.

## CONCLUSION

As PAC programs are expanded to include misoprostol, these services have the potential to become available closer to where women live and offered by a lower cadre of providers, which in turn will save lives. However, as these efforts are expanded, training providers to ensure that they give PAC clients accurate information on return to fertility, unbiased youth-friendly comprehensive contraceptive counseling, and contraceptive method provision is still critical and should be reinforced to improve access and care. Increasing the use of long-acting methods among PAC clients who want these methods should also be a part of these efforts going forward.

## Notes

**Peer Reviewed**

**Funding:** This project was funded by the William and Flora Hewlett Foundation.
